# Pharmacokinetics of Different Tacrolimus Formulations in the Early Post-Liver Transplant Period: A Scoping Review

**DOI:** 10.3390/pharmaceutics17050619

**Published:** 2025-05-06

**Authors:** Paloma Barriga-Rodríguez, Marta Falcón-Cubillo, Marta Mejías-Trueba, Pablo Ciudad-Gutiérrez, Ana Belén Guisado-Gil, Miguel Ángel Gómez-Bravo, Manuel Porras-López, María Victoria Gil-Navarro, Laura Herrera-Hidalgo

**Affiliations:** 1 Department of Pharmacy, Virgen del Rocío University Hospital, 41013 Seville, Spain; palbarrod@gmail.com (P.B.-R.); falconmarta29q@gmail.com (M.F.-C.); pablo.ciudad.sspa@juntadeandalucia.es (P.C.-G.); anab.guisado.sspa@juntadeandalucia.es (A.B.G.-G.); mariav.gil.sspa@juntadeandalucia.es (M.V.G.-N.); laura.herrera.sspa@juntadeandalucia.es (L.H.-H.); 2 Institute of Biomedicine of Seville (IBiS), Virgen del Rocío University Hospital, Consejo Superior de Investigaciones Científicas (CSIC), University of Seville, 41013 Seville, Spain; 3 Department of Hepatobiliary-Pancreatic Surgery, General and Digestive Surgery Service, Institute of Biomedicine of Seville (IBiS), Virgen del Rocío University Hospital, Consejo Superior de Investigaciones Científicas (CSIC), University of Seville, 41013 Seville, Spain; miagbravo@gmail.com; 4 Department of Intensive Care, Virgen del Rocío University Hospital, 41013 Seville, Spain; manuel.porras.sspa@juntadeandalucia.es

**Keywords:** tacrolimus, liver transplantation, pharmacokinetic, early post-liver transplant period, therapeutic drug monitoring

## Abstract

**Background:** Tacrolimus (TAC) is the cornerstone of immunosuppression after liver transplantation (LT). TAC has a narrow therapeutic index and high inter- and intra-individual pharmacokinetic (PK) variability, requiring dose individualization. This variability is more noticeable in the early post-LT period. **Objectives:** This study aimed to compare the PK of different TAC formulations in the early post-LT period and describe the main PK characteristics and plasma levels obtained with each TAC formulation used. **Methods:** The search was conducted in MEDLINE (PubMed) and EMBASE in accordance with PRISMA-ScR guidelines. The main inclusion criteria were clinical trials and observational studies focusing on the PK parameters of TAC in LT recipients during the first month post-transplant. **Results:** A total of 2169 articles were identified, of which 23 met the inclusion criteria. Various PK parameters were analyzed after LT for the different TAC formulations: intravenous (iv) and oral forms, such as immediate-release (IRT), prolonged-release (PRT), and extended-release (LCPT) formulations. PK variability was higher in the initial days after LT, with different TAC exposure between formulations. IV TAC allows the rapid attainment of therapeutic levels, but it has fallen into disuse. Regarding oral formulations, IRT reaches target levels faster than PRT and LCPT. PRT and LCPT exposure seem more stable during the first month post-LT than when using IRT. **Conclusions:** TAC formulations exhibit relevant differences in their PK profile in the early post-LT period. PK differences might influence the dose regimen and the time to achieve PK targets. Given these variations, therapeutic drug monitoring (TDM) is essential for optimizing treatment.

## 1. Introduction

Tacrolimus (TAC), a calcineurin inhibitor used worldwide as an immunosuppressant drug, is a first-line treatment in most solid organ transplants (SOTs) [1]. TAC, also known as FK-506, has been available since 1994 for the prophylaxis of liver transplant rejection. Currently, TAC is considered the primary immunosuppressive agent in most SOTs, including liver, kidney, heart, and lung transplants. The standard initial immunosuppressive regimen for transplant recipients typically combines TAC with mycophenolate mofetil (MMF) and corticosteroids to achieve adequate immunosuppression while minimizing the risk of rejection and adverse effects [1,2].

TAC is available in several formulations, including intravenous (iv) and oral forms. Among oral formulations, there are immediate-release formulations (IRT), which are administered twice daily, and prolonged-release (PRT) and extended-release (LCPT) formulations, which are both administered once daily. The once-daily formulations were specifically developed to improve patient adherence to therapy and reduce intrapatient variability in drug exposure. These formulations differ in their drug release mechanisms and should not be considered as directly interchangeable, as switching between them may affect TAC exposure and require dose adjustments [3]. Although each formulation has its own peculiarities and/or pharmacokinetic (PK) characteristics that differentiate them from one another, all of them are used in SOTs.

TAC is characterized by a narrow therapeutic index and significant inter- and intra-individual PK variability, requiring dose individualization through therapeutic drug monitoring (TDM) [2]. The key PK characteristics of TAC include its low and variable oral bioavailability (ranging from 5% to 93%) and its extensive binding to erythrocytes (85% to 95%) [4,5]. In plasma, approximately 99% of TAC is bound to plasma proteins, including α1-acid glycoprotein and albumin, with a small fraction bound to lipoproteins [1,5]. Furthermore, TAC is extensively metabolized by cytochrome P450 (CYP) 3A isoenzymes in both the liver and the gastrointestinal tract [4]. It exhibits high lipophilicity, low clearance (Cl), and is predominantly excreted via the biliary route, with around 95% of its metabolites eliminated using this route. The terminal elimination half-life (t1/2) of TAC varies significantly, ranging from 4 to 41 h [1].

The PK of TAC is influenced by several factors, including individual patient characteristics, such as the type of organ transplantation, age, CYP3A5 genotype, hematocrit (HCT) and albumin (ALB) concentrations, aspartate aminotransferase (AST) levels, and whether TAC is administered with food or if the patient experiences diarrhea. Additionally, drug-related factors, particularly the formulation type, play an essential role in TAC PK. Finally, intervention-related factors, such as the time elapsed since transplantation, the concomitant use of corticosteroids, and potential drug interactions, also significantly impact TAC PK [2,5,6].

In liver transplantation (LT) specifically, dose control is expected to be more complex due to the drug’s PK peculiarities, the function of the transplanted organ, and patient-specific idiosyncrasies. Moreover, since TAC is primarily metabolized via the hepatic route, its PK is differentially affected across patients by factors such as the regeneration of the transplanted organ over time, the organ’s functional state, and even the genotypic/phenotypic profile of the donor’s metabolizing enzymes [7].

TAC PK is highly variable and more difficult to control in the early post-transplant period due to factors such as impaired liver function affecting TAC clearance, clinical instability, and the concomitant use of corticosteroids during this time [2,8]. In the first few days, TAC absorption and excretion may be altered due to impaired gastrointestinal function, resulting in lower and more variable oral bioavailability (F) [9]. In adult patients, early within-patient variability (between days 8 and 30) has been associated with long-term graft and patient survival, and complications such as nephrotoxicity, cardiotoxicity, and neurological adverse events. This highlights the importance of early interventions to reduce variability [1]. In the long term, once the patient stabilizes, TAC PK becomes more predictable. Therefore, close monitoring of TAC during the first days after LT is crucial.

The aim of this scoping review is to compare the exposure and PK of different TAC formulations in the early post-LT period and describe the main PK characteristics and plasma levels achieved, depending on the TAC formulation used.

## 2. Methods

A scoping review was conducted in accordance with the PRISMA-ScR (Preferred Reporting Items for Systematic reviews and Meta-Analyses extension for Scoping Reviews) guidelines [10].

### 2.1. Eligibility Criteria

The inclusion criteria were defined according to the Population, Intervention, Comparison, Outcome, and Study design (PICOS) process as follows:

- Population: LT recipients (>18 years old) during the first month post-transplant.
- Intervention: Immunosuppressive treatment with TAC (despite the combination with other immunosuppressant drugs).
- Comparison: With (different formulations of TAC) or without a comparator.
- Outcomes: PK parameters and/or plasma concentrations of TAC.
- Study design: Clinical trials and observational studies.
- All articles that did not meet the inclusion criteria were excluded:
- Studies focused exclusively on the pharmacogenetics of TAC.
- Studies in which TAC was not administered via the systemic route.
- Studies in which the type of TAC formulation used could not be identified.

### 2.2. Data Source and Search Strategy

The search was conducted in two databases, MEDLINE (PubMed) and EMBASE, using controlled vocabulary and covering studies published up to 15 August 2024. In order to conduct the search and minimize potential publication bias, all references of the retrieved articles were carefully examined, allowing for the identification of relevant studies that may not have been detected during the initial review.

A search strategy was defined based on the proposed PICOS question and is detailed in Table 1.

### 2.3. Study Screening and Selection

First, duplicate articles were removed. Then, two reviewers (MFC and PBR) independently read the titles and abstracts of the articles. Documents that met the inclusion criteria and those that did not provide sufficient information to determine their exclusion were selected. Next, the full texts of the articles selected for the review were read. Finally, a critical review of the selected full-text articles was carried out.

To ensure reproducibility and minimal bias, all discrepancies were resolved through discussion and consensus with other reviewers (LHH and MMT).

### 2.4. Data Extraction

The reviewers PBR and MFC independently extracted data, while MMT and LHH examined all extraction sheets to ensure accuracy. Direct communication was established with the authors to obtain details not included in the published reports.

A descriptive analysis of the main characteristics of the included studies was performed, in which different variables were extracted and presented in tables. The design and target population of each identified study were compiled in Table 2, including the following information:

- Author and year of publication.
- Study design.
- Population: number of subjects, sex, age, and ethnicity.
- Main objective of the study.
- Primary variable of interest.
- Immunosuppressive regimen.
- Follow-up period.
- Key PK findings.

Additionally, Table 3, based on the TAC formulation, summarizes the following data:

- TAC regimen and time to first dose post-LT.
- Time to PK analysis.
- PK target range, as concentration or area under the curve (AUC).
- TAC exposure:
  - Concentrations: Minimum concentration (Cmin), which is the concentration immediately prior to dose administration, the steady-state concentration (Css), or the maximum concentration (Cmax).
  - AUC: A measure of bioavailability, represents the total amount of the drug that reaches systemic circulation.
- PK parameters: Related to absorption, absorption rate constant (Ka), absolute bioavailability (F), time to maximum plasma concentration (Tmax); related to distribution, apparent volume of distribution (Vd); and related to elimination clearance (Cl) or elimination half-life (t1/2).

### 2.5. Quality Assessment

Although this section is not mandatory for scoping reviews, a search was conducted to identify quality assessment tools applicable to the papers included in our review. However, due to the heterogeneity of qualitative methods used in the identified studies, quality assessment was not feasible, as no standardized tool was found to evaluate them uniformly.

## 3. Results

### 3.1. Search Results

A total of 2167 articles were identified, 2165 of which were retrieved from the consulted databases (524 from PubMed and 1643 from EMBASE), while 2 were identified through reference and citation searches of the included papers. After removing duplicates using Mendeley, 1783 articles remained.

**Figure 1:**
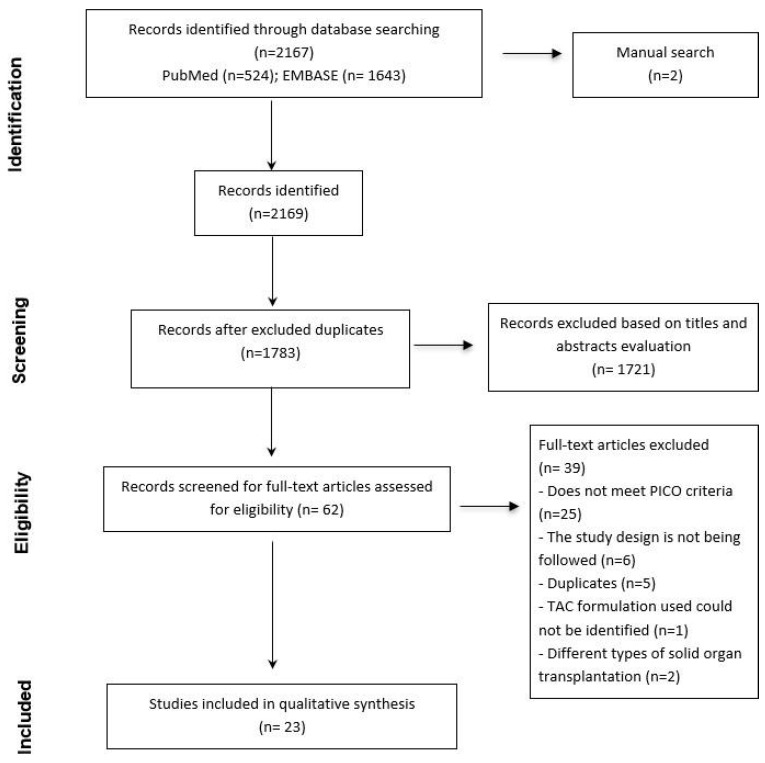
Study selection flowchart.

Based on the evaluation of titles and abstracts, 1721 articles were further excluded. The remaining 62 potentially relevant studies were reviewed in full text, of which 39 were excluded before data extraction. Ultimately, 23 studies met the inclusion criteria and were included in this scoping review (Figure 1).

### 3.2. Characteristics of the Included Studies

Table 2 and Table 3 present the variables and results of the articles included in this review. As summarized in Table 2, among the 24 included studies, 8 were randomized clinical trials [2,13,16,19,23,26,27,28], 1 was a non-randomized clinical trial [20], and 2 were sub-studies derived from clinical trials [9,22]. Of the remaining studies, four were retrospective [15,18,21,25], six were prospective [8,11,12,14,17,29], and two applied a mixed methodology, incorporating both retrospective and prospective data collection [7,24].

The main variable in most studies was the measure of TAC exposure, given as concentration and/or the AUC, except in four studies [13,19,28,29], where other variables were prioritized. The follow-up period varied considerably across studies, ranging from short-term assessments (3–6 days post-LT) to long-term evaluations extending up to 5 years.

### 3.3. Characteristics of Tacrolimus Treatment and Pharmacokinetic Analysis

Among the 23 included studies, TAC was administered iv in 1 study [11], while 4 studies used both iv and oral administration [14,17,20,23]. The remaining studies administered TAC exclusively via the oral route. TAC dosage was based on body weight and depended on the TAC formulation, as shown in Table 3. The initial dose was administered within the first 24 h post-LT in most studies, except in one study [27], where TAC was initiated between postoperative days (PODs) 1 and 3.

All studies included PK evaluations within the first week following LT. Additionally, as summarized in Table 2, PK data were collected at various time points throughout the first month post-LT. Of the 23 studies analyzed, 3 did not specify a PK target [11,18,25]. In contrast, the PK target was Cmin in 19 studies, while 1 study employed the AUC_0–24h_ as the target parameter [2]. In two studies, Cmin targets were stratified according to PODs [8,24].

Population PK models were developed in four studies [2,7,11,26]. Among these, two studies best described TAC PK using a one-compartment model [2,7], while two studies employed a two-compartment model [11,26]. In one study, the final model was refined by incorporating AST, ALB, HCT, and PODs as covariates (Covs) [7].

### 3.4. Pharmacokinetics Outcomes

Various PK parameters were described after LT for the different TAC formulations (Table 3):

#### 3.4.1. Intravenous Formulation

Five studies analyzed the PK of IV TAC [11,14,17,20,23] within the first week post-LT. The Css target in those studies oscillated between 10 and 20 ng/mL. The reported Css values ranged from a minimum of 8.15 ng/mL [11] to a maximum of 16.6 ng/mL [17]. Two studies analyzed the AUC_0–24h_ during the first postoperative week, reporting values of 369.94 ng·h/mL [14] and 399 ng·h/mL [17].

#### 3.4.2. Immediate-Release Formulations

Among the studies included, 12 analyzed the PK of IRT formulations [2,7,8,12,13,15,16,22,23,24,27,28], and all studies described Cmin achieved during the first month after LT. The Cmin target in those studies oscillated between 5 and 20 ng/mL. Three studies [13,16,22] reported the Cmin values obtained on day 1 after LT, which range between 8.98 and 9.6 ng/mL for a dose of 0.1–0.15 mg/kg/day. One of them [13] compared oral and nasogastric administration of IRT, and Cmin after nasogastric administration was remarkably higher (Cmin 11.6 ng/mL). One study [22] reported an increase in Cmin on day 3 vs. days 1, 7, and 14 [Cmin (day 3) 10.41 ng/mL vs. Cmin (days 1, 7, 14) 7.43–9.18 ng/mL]. Between day 7 and a one-month post-LT, Cmin values were generally between 8 and 9 ng/mL [2,8,13,16,22,23,24,27], except for one study [28], which administered a fixed dose and reported lower values (Cmin 4 ng/mL).

Cmax during the initial days was lower compared to values reported in the second week, increasing from 12.21 ng/mL on day 1 to 29 ng/mL by week 2. By week 3, Cmax values returned to levels comparable to those observed in the first postoperative week [16,18,22,23,24,27].

AUC measurements varied across studies. The AUC_0–6h_ was evaluated in one study [12], and the AUC_0–12h_ in four studies [2,8,12,22]; one of them [2] established a target AUC_0–12h_ between 120 and 150 ng/mL/h. During the first week, the AUC_0–12h_ was 82.87 ng·h/mL on day 1, increasing to 135–150 ng·h/mL by day 7. This range was maintained during week 2 but decreased to 110 ng·h/mL in week 3. The AUC_0–24h_ was reported in five studies [16,22,23,24,27], with values on day 1 ranging from 135.62 to 263.82 ng·h/mL. During weeks 1 and 2, the AUC_0–24h_ ranged from 241.22 to 283.19 ng·h/mL, which decreased in week 3 to 220–239.3 ng·h/mL.

#### 3.4.3. Prolonged-Release Formulations

A total of 17 studies analyzed the PK of PRT formulations [2,9,13,14,16,17,18,19,20,21,22,23,24,25,26,28,29]. Cmin was analyzed in 14 studies [2,9,13,14,16,18,20,21,22,23,24,25,28,29]. Cmin target oscillated between 5 and 20 ng/mL. Five studies reported Cmin obtained on day 1 [9,13,16,19,22]. Cmin values for oral administration on day 1 range between 2.38 and 9.97 ng/mL. In those studies, doses varied between 0.1 and 0.2 mg/kg/day. The Cmin values on day 1 reported after nasogastric administration were 5.27 and 9.4 ng/mL for doses of 0.2 mg/kg/day [13].

The reported Cmin between days 2 and 7 varied within 6 and 12 ng/mL [2,13,18,20,22,23,25,29], except for two studies [25,28], where doses were not normalized by weight, and in which the reported Cmin was lower than the rest (Cmin 2.42–2.36 ng/mL). From the end of the first week to the first month, Cmin values were generally maintained between 7 and 10 ng/mL. In this period, the highest Cmin reported was 15 ng/mL [2]; in this study, the dose varied between 0.1 and 0.3 mg/kg/day, and IV TAC was administered first. In one study [28], Cmin was the lowest, around 3 ng/mL, with an initial dose not normalized by weight (1 mg) and a Cmin target of 5 ng/mL.

Cmax was reported in seven studies [9,14,16,20,22,23,24]. On day 1, Cmax values ranged from 10.59 to 21.29 ng/mL, with similar values observed on day 7 (13 to 23 ng/mL). By weeks 2 and 3, Cmax increased slightly, ranging from 25 to 26 ng/mL.

Eight studies assessed the AUC_0–24h_ [2,9,14,16,17,20,22,23]. In one study [2], a target AUC_0–24h_ range was established (240–300 ng/mL/h). On day 1, the AUC_0–24h_ values ranged from 145.97 ng·h/mL [16] to 374.8 ng·h/mL [17]. By day 3, the values increased to between 300 and 450 ng·h/mL. On day 7, the range narrowed to 235–360 ng·h/mL. During weeks 2 and 3, the AUC_0–24h_ values remained relatively stable between 300 and 400 ng·h/mL.

#### 3.4.4. Extended-Release Formulations

The PK of the LCPT formulation was analyzed in three studies [25,27,29]. Cmin was obtained in all of them, with a target that oscillated between 5 and 20 ng/mL. During the first week post-LT, Cmin values increased from 3.22 ng/mL on day 1 [27] to 5.05 ng/mL on day 3 [25], reaching 7 to 8 ng/mL by day 7 [25,27,29]. This range of 7 to 9 ng/mL was maintained in subsequent measurements.

Cmax was reported in one study [27], with a value of 5.95 ng/mL on day 1. During weeks 1 and 2, Cmax ranged between 17 and 21 ng/mL.

The AUC_0–24h_ was assessed in one study [27]. On day 1, the AUC_0–24h_ was 68.18 ng·h/mL, increasing to 251.29 ng·h/mL in week 1 and 279.59 ng·h/mL in week 2.

## 4. Discussion

This scoping review identified distinct TAC exposure and PK characteristics among different TAC formulations and variability within the same formulation during the early post-LT period. IRT was the most extensively studied formulation, with Cmin being the primary variable analyzed.

The administration of iv TAC in the first days post-LT has been shown to facilitate the attainment of therapeutic TAC-C [11,14,17,20,23]. Reduced gastrointestinal motility, commonly observed in LT recipients immediately after surgery, may limit TAC bioavailability [9]. The initial iv TAC dose helps mitigate the risk of underdosing [20,23]. In clinical practice, the IV formulation is outdated due to the higher incidence of adverse effects and the difficulty of monitoring plasma levels compared to oral formulations.

Nasogastric administration on the first post-LT day has also been investigated and has been associated with higher TAC levels [9,13]. When comparing oral and nasogastric administration of IRT and PRT on day 1 post-transplant, Cmin was higher with nasogastric administration for both formulations [13]. This suggests that nasogastric administration may serve as an effective alternative for patients who cannot tolerate the oral route, achieving therapeutic TAC levels during the early post-LT period.

Dosage adjustments are necessary in the days following LT due to the significant inter- and intra-individual variability in TAC exposure, which differs between the first day, the first week, and the remainder of the post-LT period. Thus, individualized dosing through TDM is crucial and should be performed daily during the early post-LT period, regardless of the TAC formulation used. While the AUC is considered the PK parameter most closely associated with clinical outcomes, Cmin is widely used for routine TDM of TAC in most transplant centers [1]. Depending on the immunosuppressive regimen used, the target Cmin may vary. When combined with MMF and CS, TAC is typically targeted to a Cmin of 6–10 ng/mL during the first 4 weeks post-LT. In contrast, TAC monotherapy or a corticosteroid-free regimen may require a higher Cmin target of 10–15 ng/mL during the first 3 months post-LT [1].

In line with the above, monitoring the evolution of factors that can affect a drug’s exposure in an individual Cov, in the first days post-LT, is essential for assessing the association with liver function and Cmin measurements [15]. Several authors have investigated the impact of these factors as covariates (Covs) in population pharmacokinetic models Covs on the PK of TAC during the early post-LT period. Oteo I. et al. [7] developed a PK model for IRT that incorporated multiple Covs, such as AST, ALB, HCT, and PODs, as predictors of TAC PK variability during the first two weeks post-LT. Moreover, Cl was found to increase with PODs within the first 30 days post-LT [30]. In contrast, Allard M. et al. [26] initially included age, sex, weight, mean body mass index, HCT, glomerular filtration rate, GPT, AST, ALB, and BIL in their initial PK model for IRT, which included both early and stable LT recipients. However, none of these Covs were retained in the final model, as they were not significantly associated with Cl. Covs seem to be particularly useful in the first days post-LT, when significant hepatic alterations and clinical instability in patients might be useful in predicting TAC concentrations. Once patients are stabilized, these Covs no longer significantly improve TAC concentration predictions using those PK models.

TDM is also influenced by the TAC formulation. In studies where neither iv nor nasogastric TAC was used, higher initial oral doses were required to overcome early postoperative underexposure [13]. The required dose varies depending on the TAC formulation, with PRT generally requiring higher mean doses than IRT to achieve comparable systemic exposure [16,23]. Moreover, the variability in TAC-C was higher for PRT than IRT between days 1 to 7 post-LT. However, the linear relationship between the AUC_0–24h_ and Cmin was found to be similar for IRT and PRT [23], as noted by Coilly A et al. [31]. Ericzon BG et al. [22] confirmed that the established TDM system for IRT could also be applied to PRT, though a higher starting dose of PRT was required to achieve similar systemic exposure. Additionally, Iwasaki M. et al. [24] observed that while Cmin values were nearly equivalent between the formulations, the AUC_0–24h_ tended to be higher for PRT. This higher AUC_0–24h_ was consistent with the use of higher doses, as it can indicate greater TAC exposure.

Given these findings, PRT and IRT may not share the same Cmin targets during the early post-LT period. Although none of the reviewed studies explicitly differentiated Cmin targets based on TAC formulation, two studies [8,24] established distinct Cmin targets according to PODs. Additionally, Brunet M et al. [1], in their second consensus report, proposed different Cmin targets based on the immunosuppressive regimen used and PODs; however, no studies specifically applied this distinction. The Cmin target in the early post-LT period should consider the immunosuppressive regimen used; the PODs, which correlate with greater or lesser clinical instability; and, especially, the patient’s liver function and the TAC formulation used.

In this context, LCPT emerges as a formulation with distinct PK advantages. LCPT exhibits higher bioavailability than both PRT and IRT [1]. During the first month post-LT, LCPT achieved therapeutic trough levels earlier than PRT, despite requiring a 25% lower median dose [25]. DuBay DA et al. [27] compared LCPT and IRT, finding that on day 1, the AUC_0–24h_ and Cmin were lower for LCPT but became similar by day 7. By day 14, the AUC_0–24h_ was higher for LCPT, while Cmin remained comparable. Similarly, Bilbao I et al. [29] compared LCPT and PRT following 3 to 5 days of treatment with IRT. At week 1, Cmin was higher with LCPT than with PRT (6.6 ng/mL vs. 5.8 ng/mL). However, comparable Cmin levels were observed throughout the remainder of the follow-up period. Notably, LCPT achieved similar TAC-C with a lower total daily dose and exhibited less fluctuation in drug levels [1].

In summary, comparing the different TAC formulations analyzed in this scoping review, iv TAC reaches higher concentrations, potentially increasing the risk of adverse effects; therefore, it is outdated in clinical practice. On day 1, Cmin is the highest for IRT and the lowest for LCPT, which may support the use of IRT in the early post-transplant period to achieve therapeutic levels, followed by a transition to PRT or LCPT. From week 1 to month 1, Cmin remains similar across the three oral formulations studied. The AUC_0–24h_ obtained with PRT is higher than that observed with IRT or LCPT, which may justify the need for different Cmin targets depending on the TAC formulation used. Moreover, Tmax values of up to 12 h and a Cmax of 5.95 ng/mL on day 1 with LCPT align with the observed Cmin data in the early post-LT period. From day 7 onward, LCPT maintains Cmin levels within the therapeutic range with lower variability compared to PRT and IRT.

This review provides a comprehensive summary of the available evidence on the PK of TAC during the early post-LT period, incorporating data from observational studies and clinical trials. However, several limitations should be considered: the included studies focus on a limited post-transplant period; no data were identified regarding the specific therapeutic Cmin target for different TAC formulations or immunosuppressive regimens; and only two studies have examined the PK of LCPT, limiting the reliability of the conclusions for this formulation. Further research is needed to evaluate the administration of an initial dose via nasogastric tube and its potential role in achieving therapeutic levels, as none of the included studies addressed this approach with LCPT.

## 5. Conclusions

This scoping review provides a comprehensive overview of the PK of different TAC formulations. IV TAC has been used in the early post-LT period to achieve therapeutic levels more rapidly. However, its use has declined due to the complexities associated with its management compared to oral TAC formulations. As a potential alternative, nasogastric tube administration may be considered when the oral route is unavailable, although this approach has been evaluated in only two studies, neither of which included LCPT.

If the patient is successfully extubated, IRT reaches optimal levels most rapidly. However, its twice-daily dosing schedule may pose adherence challenges. Among the extended-release formulations, PRT achieves therapeutic levels earlier than LCPT, though LCPT requires a lower total daily dose and shows reduced variability in therapeutic levels by day 7. Given the PK variability observed among the different TAC formulations, as well as inter- and intra-individual variability, it is essential to implement appropriate TDM methods.

## Figures and Tables

**Table 1:** Detailed search strategy.

Database	Search Strategy
PubMed	((“liver transplantation*”[MeSH Terms] OR “liver transplantation*”[Title/Abstract] OR “liver transplant*”[Title/Abstract] OR “liver transplant recipient*”[Title/Abstract]) AND (“tacrolimus/pharmacokinetic*”[MeSH Terms] OR “population pharmacokinetic*”[Title/Abstract] OR “Drug Monitoring”[MeSH Terms] OR “Drug Monitoring”[Title/Abstract]) AND (“Tacrolimus”[MeSH Terms] OR “Tacrolimus”[Title/Abstract]))
EMBASE	(‘liver transplantation’/exp OR ‘liver transplantation’:ab,ti OR ‘liver graft’/exp OR ‘liver graft’:ti,ab) AND (‘tacrolimus’/exp OR ‘tacrolimus’:ab,ti) AND (‘pharmacokinetics’/exp OR ‘pharmacokinetics’:ab,ti OR ‘population pharmacokinetics’:ab,ti OR ‘drug monitoring’/exp OR ‘drug monitoring’:ab,ti)

* includes additional endings, which can affect the search results.

**Table 2:** Characteristic of the included studies.

Author, Year	Study Design	Population (N° Subjects, Sex, Age * and Ethnicity)	Main Objective	Primary Variable	IS Regimen	Follow-Up Period (Post-LT)	Key PK Findings
Jain AB., 1993 [11]	Prospective study	N = 9 (8 males) Age: 24–64	To characterize the iv TAC PK during the immediate PODs and to compare actual and predicted TAC-Css. To develop a popPK model.	Css	TAC + CS	3 to 6 days, until the last dose of iv TAC	- Two-compartment model without covariables best describes TAC PK profile. - Liver function highly influences TAC-Css. - Time until steady stage using continuous infusions is long (45 h) due to TAC’s extended half-life.
Cantaro vich M., 1998 [12]	Prospective study	N = 9 (4 males) Age: 55 ± 9	To determine a single time point TAC measurement that predicts AUC after LT.	Cmin	TAC + CS + AZA	Until 12 weeks	- Correlation of C2, C3, or C4 and the AUC_0–6h_ suggests that it could be considered for TAC TDM and should be correlated with the AUC_0–12h_.
Truneča P., 2010 [13]	Multicenter, phase III, randomized, double-blind, double-dummy, parallel-group, comparative CT	N = 471 (331 males) IRT: 234, PRT: 237 Age: - IRT: 52.8 ± 9.5 - PRT: 52.7 ± 9.1 Caucasian (458), others (12)	To compare the efficacy and safety of IRT and PRT.	The event rate of local biopsy-proven acute rejection within 24 weeks post-LT	TAC + CS ± MMF (when acute rejection)	12 months	- Initial dose of PRT should be higher than IRT to achieve similar exposure and to avoid underexposure in early PODs. - TAC levels on day 1 were higher after nasogastric than oral administration.
Sugawar a Y., 2011 [14]	Prospective study	N = 12 (5 males) PK profile: 9 subjects Age: 49 (37–61)	To determine the safety and tolerability of OD TAC.	Cmin	TAC + CS	No data	- OD TAC shows high correlation between TAC exposure (AUC0–24 h) and levels (Cmin). - OD regimen improves compliance and maintains immunosuppression.
Oteo I., 2011 [15]	Retrospective study	N = 75	To address the inherent difficulties in empirical TAC TDM by rationally integrating the sources of variability between Cmin and Cmin/dose.	Cmin	TAC + CS ± AZA	Until 15 days	- Bayes prediction based on a population model is the optimum methodology because it does not require reaching SS and provides individual PK parameters with only one value of Cmin after dose.
Fischer L., 2011 [16]	Phase II, randomized, open-label, prospective clinical trial	N = 129 (94 males) PRT: 67, IRT: 62 (PK profile: 77) Age: - PRT: 49.4 (24–65) - IRT: 52.4 (27–68) Caucasian (126), Others (3)	To evaluate and compare PK parameters of PRT and IRT following 1st administration and under SS conditions.	AUC_0–24h_	TAC + CS ± MMF ± AZA	6 weeks	- AUC_0–24h_ on day 1 for PRT was 50% lower than IRT at equivalent doses. On day 4, AUC_0–24h_ was similar for both formulations (IRT and PRT). - Initial dose of PRT should be higher than IRT to achieve similar exposure.
Yano I., 2011 [8]	Prospective study	N = 14 (8 males) Age: 58 ± 6	To evaluate the relationship between blood TAC-C at each sampling time and drug exposure during the dosing intervals.	Cmin	TAC + CS	3 weeks	- C2, C4, and C8 TAC levels correlate better with AUC_0–12h_ than trough levels. - Rejection risk in the first 10 days post-LT is linked to average trough TAC levels on PODs 2–4.
Oteo I., 2012 [7]	Retrospective and prospective study	N = 75 (development) N = 10 (validation) N = 15 (applicability)	To develop a population PK model for TAC in the first 2 weeks post-LT, and to estimate individual PK to demonstrate its applicability for dose individualization.	Cmin	TAC + CS ± AZA	Until 15 days	- One-compartment model best describes TAC PK profile. - AST, ALB, HCT, and PODs are key predictors of TAC PK variability in early post-LT PK model development. - High variability in TAC t_1/2_ explains delayed SS in some patients.
Mita A., 2014 [17]	Prospective study	N = 10 (4 males) Age: 44.9 ± 16	To determine the optimal initial dose of orally administered PRT following iv TAC after LT.	AUC_0–24h_	TAC + CS	No data	- Interpatient variation in absorption may affect PRT more significantly than IRT. - Optimal PRT dose is 8 times the iv TAC dose.
Gastaca M., 2015 [18]	Retrospective study	N = 150 Age: 55.5 (20–67)	To describe the long-term efficacy of PRT.	Cmin	TAC ± MMF ± CS ± antibody	5 years	- Long-term immunosuppression with PRT in LT is effective.
Truneča P., 2015 [19]	Multicenter, phase IIIb, randomized, open-label, parallel-group clinical trial (DIAMOND trial)	N = 844 (594 males) Age: 53.7–54.3 ± 10.6–9.1 Black/African (15), Asian (5), other (25)	To determine whether delaying PRT until day 5 or reducing its initial dose improves renal function versus to an immediate post-LT dose.	Renal function estimated by eGFR at week 24	TAC + MMF ± BAS ± CS	24 weeks	- Target TAC-C was achieved within 48 h after PRT initiation in all groups.
Song GW., 2016 [20]	Phase IV, non-randomized, prospective, open-label pilot study	N = 11 (9 males) (PK profile: 10) Age: 51.6 ± 5.8 Asian	To examine the PK of PRT after the 1st oral dose and under SS conditions (before the 10th oral dose).	AUC_0–24h_ days 6 and 14	TAC + MMF + CS ± BAS	12 weeks	- IV TAC in the first days post-LT prevents underexposure due to reduced gastrointestinal function. - AUC_0–24h_ and Cmin correlation was stronger after the 2nd PRT dose than at SS.
Más-Serrano P., 2017 [21]	Retrospective study	N = 99 (83 males) Age: 57	To analyze the efficacy and safety of PRT individualized dosing through a Bayesian approach.	Cmin	TAC + MMF + CS	4 years	- Target TAC-C was achieved within 48 h after PRT initiation in 75% of patients.
Ericzon BG., 2017 [22]	Substudy of a phase III, double-blind, randomized clinical trial	N = 25 (19 males) Age: - PRT: 53.4 - IRT: 55.7 Caucasian	To compare AUC_0–24h_ of TAC between PRT and IRT.	AUC_0–24h_	TAC	2 weeks	- AUC_0–24h_ on day 1 for PRT was lower than IRT with the same dose (0.1 mg/kg/day). With a higher dose (0.2 mg/kg/day), PRT exposure was greater than IRT. - Initial dose of PRT should be higher than IRT to achieve similar exposure. - TDM strategies are valid for both formulation (IRT and PRT).
Shin MH., 2018 [23]	Phase IV, randomized, open-label, controlled, comparative clinical trial	N = 100 (84 males) (PK profile: 86) Age: - PRT: 53.8 ± 7.6 - IRT: 50.6 ± 7.7 Asian/Oriental	To evaluate the PK of PRT and IRT under early and SS conditions.	AUC_0–24h_ on days 6 and 21	TAC + CS ± MMF ± BAS	24 weeks	- PRT and IRT show a similar correlation between AUC_0–24h_ and Cmin. - Initial dose of PRT should be higher than IRT to achieve similar exposure. - IV TAC in the first days post-LT prevents underexposure.
Iwasaki M., 2018 [24]	PRT: prospective study; IRT: retrospective study	N = 22 (13 males) PRT: 9, IRT: 13 Age: - PRT: 51 ± 9 - IRT: 58 ± 6	To investigate the PK of PRT with simultaneous measurements of blood TAC-C in the early stage after LT and compare with previous IRT data.	Cmin	TAC + MMF + CS	3 weeks	- PRT daily maintenance dose was nearly double that of IRT, though C_0_ levels were similar, and AUC_0–24h_ for PRT tended to be higher than IRT. - PRT and IRT may require different C_0_ targets in early post-LT period due to higher systemic exposure with PRT.
Baccaran i U., 2019 [25]	Retrospective study	N = 35 (27 males) LCPT: 16, PRT: 19 Age: - LCPT: 59 ± 9 - PRT: 59 ± 8	To compare LCPT and PRT in terms of therapeutic trough levels and daily dosage after LT.	Cmin	TAC + CS	30 days	- Target TAC-C was achieved in the 1st month earlier for LCPT than PRT, despite a 25% lower median dose for LCPT. - LCPT dose compared to PRT dose may be reduced to reach target TAC-C.
Allard M., 2019 [26]	Multicenter, randomized, prospective clinical trial (CONVERSI ON trial)	N = 90 (PK profile: 24, 20 males) Age: - Group A: 57 (53–60) - Group B: 59 (54–62)	To develop a popPK model for PRT.	Cmin and AUC_0–24h_	TAC	180 days	- Two-compartment model best describes TAC PK profile.
Undre N., 2019 [9]	Substudy of the DIAMOND trial, phase IIIb, randomized, controlled	N = 11 (PK profile: 10)	To assess the absorption and PK profile of PRT when administered by nasogastric tube immediately post-LT.	Cmin and AUC_0–24h_ on days 1 and 3	TAC + MMF + CS ± BAS	3 days	- TAC absorption was not significantly altered when opened PRT capsules were administered via nasogastric tube compared to intact capsules administered orally.
DuBay DA., 2019 [27]	Multicenter, phase II, randomized, open-label clinical trial	N = 58 (40 males) (PK profile: 44) Age: 55 (21–72) Hispanic, American, Asian, Black	To analyze the PK of LCPT (AUC_0–24h_ and Cmax) and 24 h trough concentrations (C_24_) within the first 14 days post-LT, and to compare with IRT, the patients who achieved TAC-C within the first 14 days post-LT.	AUC_0–24h_, Cmax and C24	TAC + CS ± MMF	12 months	- LCPT showed the highest correlation between AUC_0–24h_ and Cmin on day 14 compared to IRT. - LCPT and IRT showed similar efficacy and safety when used de novo in LT.
Riff C., 2019 [1]	Multicenter, phase II, randomized, open-label, prospective clinical trial	N = 80	To develop a pop PK model and BE for PRT and IRT in early and late post-LT periods and to evaluate their performance in predicting TAC AUC and dose requirements.	Cmin, AUC_0–12h_ and AUC_0–24h_	TAC + CS	6 weeks ± 7 days	- One-compartment model best describes TAC PK profile. - ‘In stable post-LT, imprecision of TAC AUC and dose estimation was lower than in immediate post-LT for both PRT and IRT. - AUC_0–24h_ and Cmin correlation for IRT was stronger than PRT on day 7, and, on week 6, was stronger for PRT than IRT.
Venkata krishnan G., 2021 [28]	Randomized trial	N = 72	To compare the safety and efficacy of PRT versus IRT.	eGFR at 1, 3, and 6 months following transplant	TAC + BAS + MMF	6 months	- Cmin of PRT was significantly lower than IRT for an equivalent dose during the first month.
Bilbao I., 2023 [29]	Multicenter, prospective, observational study	N = 163 (121 males) LCPT: 87 PRT: 76 Age: 57.3 Caucasian (159)	To compare the effectiveness of LCPT and PRT,	Incidence of treatment failure **	TAC ± MMF ± CS ± BAS	48 weeks	- LCPT achieved similar blood TAC-C with a lower total daily dose than PRT.

AUC: area under curve; AZA: Azathioprine; BAS: Basiliximab; BE: Bayesian estimator; Cmin: minimum concentration; CL: clearance; CN: calcineurin; CS: corticosteroid(s), Css: steady-state plasma concentration; CT: clinical trial; eGFR: estimated glomerular filtration rate; h: hours; IRT: twice-daily immediate release tacrolimus; IS: immunosuppressive; iv: intravenous; LCPT: once-daily extended release tacrolimus; LT: liver transplant; MMF: Mycophenolato mofetil; PBMCs: peripheral blood mononuclear cells; PK: pharmacokinetic; PODs: postoperative days; PRT: once-daily prolonged release tacrolimus; OD: once-daily; SS: steady state; TAC: tacrolimus; TAC-C: tacrolimus concentration; TDM: therapeutic drug monitoring; TD: twice-daily; t1/2: half-life; Vd: volume of distribution. * Age expressed in years as mean (±SD) or median (IQR). ** Treatment failure is defined as: the presence of biopsy-proven acute rejection, loss to follow-up, death, or graft failure throughout the follow-up.

**Table 3:** Pharmacokinetic characteristics of TAC formulations in early post-LT period.

Ref	TAC Regimen and Time to First Dose Post-LT	Time to PK Analysis	PK Target: Concentration (ng/mL) or AUC (ng/mL/h)	TAC Exposure: Concentration (ng/mL) or AUC (ng/mL/h)		PK Parameters
**IV TAC pharmacokinetic studies**						
[11]	IV TAC 0.15 mg/kg/day 1st dose: 2 to 4 h post-LT	Days 3 to 6 post-LT (Until last dose iv TAC)	No data	Css: 8.15	No data	Cl: 105.6 ± 105 L/h Vd (Vss): 1252 ± 668 L t_1/2_: 15.5 ± 11.2 h
[14]	IV TAC: 2.5 mg/kg/h 1st dose: Immediately post-LT	Daily, until conversion to PRT	Css: 17–18	Css: 15.41 ± 0.67	AUC_0–24h_: 369.94 ± 16.17	Cl: 29.7 mL/h/kg
[17]	IV TAC: 1.1 ± 0.6 mg/day	Before conversion to PRT	Cmin: 15–20 (1st two weeks post-LT)	Css: 16.6 ± 2.6	AUC_0–24h_: 399 ± 53.5	No data
[20]	Day 0 to 4 iv TAC: 0.025 to 0.05 mg/kg/day. 1st dose: Day of transplant	Day 4	Css: 10–20	Css: 12.3 ± 3.7	No data	No data
[23]	Day 0 to 4 iv TAC: 0.025–0.05 mg/kg/day 1st dose: after LT	Day 5 (prior to switch)	Css: 10–15	Css: 11.3 ± 4	No data	No data
**IRT pharmacokinetic studies**						
[12]	IRT: 0.15 mg/kg/day TD 1st dose: Immediately post-LT	Cmin: Daily AUC: week 1 and 4	Cmin: 10–20	Cmin: 13 ± 4.7	AUC_0–6h_:118.4 ± 37 AUC_0–12h_:201.9 ± 55.2	CL: 32.7 ± 16 L/h
[13]	IRT: 0.1 mg/kg/day TD 1st dose: within 24 h post-LT	Days 1 and 7	Cmin: 10–20	Cmin: Day 1: 11.6 ± 8.5 (via nasogastric) or 9.6 ± 8.8 (oral) Day 7: 9.5 ± 4.5	No data	No data
[14]	IRT: 0.1 mg/kg/day TD 1st dose: Immediately post-LT	Daily, from day 0 until day 15 post-LT or day of release from hospital	Cmin: 10–20	Cmin: 13.61 (1.5–30) 14.28 (normal AST) 12.45 (elevated AST) * 1. Cmin divided for PODs, AST, ALB and HCT	No data	No data
[16]	IRT: 0.10 to 0.15 mg/kg/day TD 1st dose: Within 6–12 h post-LT	Days 1 and 14	Cmin: 10–20	Day 1: Cmin: 8.98 ± 5.9 Cmax: 19.75 ± 8.48 Day 14: Cmin: 8.53 ± 2.85 Cmax: 25.07 ± 12.13	AUC_0–24h_: Day 1: 263.82 ± 153.36 Day 14: 286.99 ± 88.03	Tmax (h): Day 1: 2.9 ± 1.8 Day 14: 1.9 ± 1.5
[8]	IRT: 0.05 mg/kg/day TD 1st dose: Within 12 h post-LT	Weeks 1 and 3	Cmin: 10–15 (PODs 1–7), 8–12 (PODs 8–14), 6–10 (after POD 15)	Week 1: Cmin: 9.8 ± 2.6 Cmax: 16 ± 5.4 Week 3: Cmin: 7.7 ± 2.7 Cmax: 12.5 ± 4.9	AUC_0–12h_: Week 1: 140 ± 38 Week 3: 110 ± 40	Tmax (h): Week 1: 2 (1–8) Week 3: 2 (0–4) Cl/F (L/h/kg): Week 1: 0.15 ± 0.11 Week 3: 0.27 ± 0.22
[7]	IRT: 0.1 mg/kg/day TD 1st dose: In the first hours post-LT	Days 0 to 15 post-LT	Cmin: 8–12	Cmin (range): 1.5–30 Cmin/Dose: 0.1–6.1 (0–3 PODs) Cmin/Dose: 0.4–21 (4–15 PODs)	No data	Ka: 4.48 h-1 Cl/F: 11.9 ± 12.77 L/h V/F: 153 ± 29.74 L * 2 Final model parameter estimates for two periods t_1/2_: 7.68–90.74 h
[22]	IRT: 0.1 mg/kg/day TD 1st dose: Within 12 h post-LT	Days 1, 3, 7, and 14 (SS conditions)	Cmin: 10–20	Day 1: Cmin: 9.18 ± 7.26 Cmax: 12.21 ± 8.7 Day 3: Cmin: 10.41 ± 7.61 Cmax: 19.47 ± 11.69 Day 7: Cmin: 7.43 ± 2.63 Cmax: 19.94 ± 12.18 Day 14: Cmin: 8.89 ± 2.24 Cmax: 29.34 ± 22.36	AUC_0–12h_: Day 1: 82.87 ± 62.63 Day 3: 161.14 ± 109.77 Day 7: 134.95 ± 60.28 Day 14: 155.54 ± 60.2 AUC_0–24h_: Day 1: 216.63 ± 132.51 Day 3: 317.9 ± 218.63 Day 7: 249.12 ± 96.4 Day 14: 283.19± 92.21	Tmax: 1–2 h Day 1: 2 h (1–12) Day 3: 1.5 h (0.5–8) Day 7: 1.5 h (0.5–6) Day 14: 1 h (1–4)
[23]	Initial iv TAC and switch to IRT (Day 5) (iv:IRT 1:4)	Days 6 and 21 (SS conditions)	Cmin: 10–15	Day 6: Cmin: 9.73 ± 2.54 Cmax: 17.4 Day 21: Cmin: 9.08 ± 3.21 Cmax: 18.8	AUC_0–24h_: Day 6: 265.8 Day 21: 239.3	Tmax (h): Day 6: 4.5 Day 21: 2.8
[24]	IRT: 0.03 ± 0.01 mg/kg/day TD 1st dose: In the morning on the day post-LT	Daily until 3 weeks after LT	Cmin: 10–15 (PODs 1–7), 8–12 (PODs 8–14), 6–10 (after POD 15)	Week 3: Cmin: 7.7 ± 2.6 Cmax: 12.5 ± 4.7	AUC_0–24h_: Week 3: 220 ± 76	Week 3: Tmax: 2 h (0–4) Cl/F: 0.267 ± 0.213 L/h/kg
[27]	IRT: 0.10–0.15 mg/kg/day TD. 1st dose: Between 1 and 3 days post-LT	Days 1, 7, and 14	Cmin: 5–20	Day 1: Cmin: 4.42 ± 2.83 Cmax: 12.7 ± 6.29 Day 7: Cmin: 7.61 ± 4.06 Cmax: 21.1 ± 8.97 Day 14: Cmin: 7.56 ± 2.64 Cmax: 22.95 ± 14.57	AUC_0–24h_: Day 1: 135.62 ± 73.92 Day 7: 245.47 ± 102.03 Day 14: 241.22 ± 79.9	Tmax (h): Day 1: 2.67 h (1–20) Day 7: 1.51 h (0.67–16.5) Day 14: 2 h (1–14)
[2]	IRT: 0.05 mg/kg/12 h 1st dose: within 6–12 h post-LT	Day 7	AUC_0–12h_: 120–150	8.2 (3.8–16.1)	AUC_0–12h_: 152.8 (60.6–260.2)	Cl/F:39.3 (20.1–75.6) L/h Vd/F: 278 (101–444) L
[28]	IRT: 1 mg	Weeks 1, 2, and 3 and month 1	Cmin: 5	Cmin: Week 1: 3.8 Week 2: 3.76 Week 3: 4.08 Month 1: 3.72	No data	No data
**PRT pharmacokinetic studies**						
[13]	PRT: 0.2 mg/kg/day 1st dose: within 24 h post-LT	Days 1 and 7	Cmin: 10–20	Cmin: Day 1: 9.4 ± 6.8 (via nasogastric) or 6 ± 5.8 (oral) Day 7: 12 ± 5.9	No data	No data
[14]	Initial iv TAC (7–20 days) and switch to PRT	PK profile 7 days after conversion to PRT	No data	Cmin: 14.63 ± 2.68 Cmax: 27.65 ± 3.76	AUC_0–24h_: 459.54 ± 76.85	F: 10.6 ± 4% Cl: 316 mL/h/kg
[16]	PRT: 0.10 to 0.15 mg/kg/day 1st dose: Within 6–12 h post-LT	Days 1 and 14	Cmin: 10–20	Day 1: Cmin: 4.21 ± 3.31 Cmax: 10.59 ± 6.26 Day 14: Cmin: 8.82 ± 3.18 Cmax: 25.65 ± 11.61	AUC_0–24h_: Day 1: 145.97 ± 103.03 Day 14: 324.19 ± 119.07	Tmax: Day 1: 5 ± 4 h Day 14: 2.8 ± 2.5 h
[17]	Initial iv TAC and switch to PRT: 8.3 ± 6.7 mg/day (iv:PRT 1:8)	Days 1, 3, and 5 during conversion TAC iv to PRT	Cmin: 15	No data	AUC_0–24h_: Day 1: 374.8 ± 65 Day 3: 369 ± 47.4 Day 5: 412.4 ± 132.1	F: 13 ± 9%
[18]	PRT: 0.15 mg/kg/day (only TAC) or 0.1 mg/kg/day (if TAC + MMF)	1st week	No data	Cmin: 7.85	No data	No data
[19]	PRT: 0.2 mg/kg/day (Arm1) 1st dose: in the morning or within 18 h post-LT	Daily for 14 days and then on days 21 and 28	Cmin: 5–15 (until day 42)	Day 1: 3.92 ± 7.42; Days 2–14: 8.48 ± 4.58 to 12.48 ± 9.43 Day 21: 8.43 ± 3.85; Day 28: 8.74 ± 3.6	No data	No data
	PRT: 0.15–0.175 mg/kg/day (Arm2) 1st dose: in the morning or within 18 h post-LT			Day 1: 2.38 ± 5.83; Days 2–14: 7.16 ± 3.96 to 10.48 ± 8.1 Day 21: 7.82 ± 3.1; Day 28: 8.43 ± 3.78		
	PRT: 0.2 mg/kg/day (Arm3) 1st dose: Day 5 post-LT			Day 6: 6.23 ± 6.08; Day 7–14: 9.33 ± 5.77 to 10.54 ± 6.7; Day 21: 9.12 ± 4.18; Day 28: 9.38 ± 4.43		
[20]	Initial IV-TAC and switch to PRT (day 5): 0.15–0.3 mg/kg/day	Cmin: week 4; PK profile: days 6 and 14	Cmin: 10–20	Day 6: Cmin: 8.7 ± 1.6 Cmax: 13.12 ± 3.1 Day 14: Cmin: 15 ± 4.4 Cmax: 26.25 ± 8.5 Week 4: Cmin: 13.5 ± 5	AUC_0–24h_: Day 6: 235.67 ± 47.9 Day 14: 423.86 ± 86	Tmax: Day 6: 2 h (1–4.1) Day 14: 3.5 h (0–16.1)
[21]	PRT: 0.15 mg/kg/day 1st dose: Within the 1st 24 h post-LT	Days 2, 7, 15, and 30	Cmin: 8–10	Cmin: Day 2: 8.9 (5.3–11.6) Day 7: 7.5 (5.4–9.7) Day 15: 8.78 (6.9–10.65) Day 30: 9.7 (8.17–11.9)	No data	No data
[22]	PRT: 0.2 mg/kg/day 1st dose: Within 12 h post-LT	Days 1, 3, 7, and 14 (SS conditions)	Cmin: 10–20	Day 1: Cmin: 9.97 ± 6.7 Cmax: 21.29 ± 10.53 Day 3: Cmin: 14.06 ± 7.11 Cmax: 27.82 ± 11.72 Day 7: Cmin: 11.06 ± 5.63 Cmax: 23.2 ± 9.83 Day 14: Cmin: 10.47 ± 4.14 Cmax: 24.85 ± 7.24	AUC_0–24h_: Day 1: 320.44 ± 186.93; Day 3: 452.06 ± 213.19; Day 7: 358.6 ± 146.62; Day 14: 353.42 ± 109.42	Tmax: 3–4 h Day 1: 3 h (1–14) Day 3: 4 h (1–13) Day 7: 3 h (1–8) Day 14: 3 h (1–6)
[23]	Initial iv TAC and switch to PRT (Day 5) (iv:PRT 1:6)	Days 6 and 21 (SS conditions)	Cmin: 10–15	Day 6: Cmin: 8.68 ± 3.35 Cmax: 16.3 Day 21: Cmin: 8.83 ± 3.61 Cmax: 25.1	AUC_0–24h_: Day 6: 257.3 Day 21: 308.2	Tmax (h): Day 6: 3.5 Day 21: 2.8
[24]	PRT: 0.15 ± 0.05 mg/kg/day 1st dose: In the morning on the day post-LT	Daily until 3 weeks after LT	Cmin: 10–15 (PODs 1–7), 8–12 (PODs 8–14), 6–10 (after POD 15)	Week 3: Cmin: 9 ± 4.5 Cmax: 22.3 ± 8.6	AUC_0–24h_: Week 3: 330 ± 103	Week 3: Tmax: 4 h (0–12) Cl/F: 0.319 ± 0.205 L/h/kg
[25]	PRT: 5.26 ± 1.91 mg/day	Days 3, 5, 7, 15, and 30	No data	Cmin: Day 3: 2.42 ± 2.75 Day 5: 4.17 ± 2.05 Day 7: 6.06 ± 3.03 Day 15: 6.69 ± 2.71 Day 30: 7.96 ± 4.16	No data	No data
[26]	Initial IRT and switch to PRT (day 7) (IRT:PRT 1:1)	Cmin: days 5, 7, and 30; PK profile: day 14	Cmin: 6–10	Cmin: 8.5 (5.4–10.2)	AUC_0–24h_: Non compartmental 251.3 (95%, CI 108.5–460.7) Model-predicted: 235.6 (95% CI, 139.6–598.7)	Cl: 5.11 (95% CI, 4.36–5.93) L/h Vc: 86.9 (95% CI, 41.1–126) L Vp: 142 (95% CI, 88.4–196) L
[9]	PRT via nasogastric 0.2 mg/kg/day or 0.15– 0.175 mg/kg/day (if TAC + BAS) 1st dose: Immediately post-LT	Days 1 and 3	Cmin: 5–15	Day 1: Cmin: 5.27 Cmax: 15.1 Day 3 Cmin: 8.77 Cmax:19.1	AUC_0–24h_: Day 1: 193 Day 3: 301	Tmax (h): Day 1: 2 (2–24) Day 3: 4.5 (0.5– 24)
[2]	PRT: 0.1 mg/kg/day 1st dose: within 6–12 h post-LT	Day 7	AUC_0–24h_: 240–300	8.6 (0.8–55)	AUC_0–24h_: 316.5 (34.5–775.2)	Cl/F: 48.7 (18.9–91.3) L/h Vd/F: 589 (335–2857) L
[28]	PRT: 1 mg	Weeks 1, 2, and 3 and month 1	Cmin: 5	Cmin: Week 1: 2.36 Week 2: 2.9 Week 3: 2.6 Month 1: 2.91	No data	No data
[29]	Initial IRT and switch to PRT (day 3 to 5) (IRT:PRT 1:1)	Weeks 1, 2, and 4	Cmin: 6–12	Cmin: Week 1: 5.8 ± 2.4 Week 2: 8.2 ± 3 Week 4: 7.8 ± 3.3	No data	No data
**LCPT pharmacokinetic studies**						
[25]	LCPT: 5.19 ± 1.72 mg/day	Days 3, 5, 7, 15, and 30	No data	Cmin: Day 3: 5.05 ± 3.58 Day 5: 7.35 ± 5.12 Day 7: 8.03 ± 5.44 Day 15: 8.62 ± 7.86 Day 30: 9.1 ± 5.78	No data	No data
[27]	LCPT: 0.07 to 0.11 mg/kg/day except if black: 0.09–0.13 mg/kg/day 1st dose: Between 1 and 3 days post-LT	Days 1, 7, and 14	Cmin: 5–20	Day 1: Cmin: 3.22 ± 2.39 Cmax: 5.95 ± 3.46 Day 7: Cmin: 7.33 ± 3.54 Cmax: 17.15 ± 7.9 Day 14: Cmin: 7.41 ± 4.17 Cmax: 21.3 ± 9.93	AUC_0–24h_: Day 1: 68.18 ± 37.4 Day 7: 251.29 ± 102.6 Day 14: 279.59 ± 139.86	Tmax (h): Day 1: 12 (1.48–24.2) Day 7: 4 (0–12) Day 14: 4 (1–16)
[29]	Initial IRT and switch to LCPT (day 3 to 5) (IRT:PRT 1:1)	Weeks 1, 2, and 4	Cmin: 6–12	Cmin: Week 1: 6.6 ± 3.4 Week 2: 8 ± 3.9 Week 4: 7.7 ± 4.4	No data	No data

ALB: albumin; ALT: alanine aminotransferase; AST: aspartate aminotransferase; BIL: bilirubin; Cmax: maximum concentration; Cr: creatinine; Css: steady-state concentration; F: bioavailability; HCT: hematocrit; Kg: kilogram; mg: milligram; PODs: postoperative days; Vss: steady-state volume of distribution. * 1. Cmin divided for PODs, AST, ALB, and HCT: Days 4–15: 12.8 (5.1–22) (normal AST, ALB and HCT); 10.8 (4.5–16.4) (normal AST, reduced ALB and HCT); 14.6 (1.8–30) (elevated AST, normal ALB and HCT); 8.5 (4.6–20.2) (elevated AST, reduced ALB and HCT). Days 0–15: 12.8 (normal AST, ALB, and HCT); 11.5 (normal AST, reduced ALB and HCT); 15.1 (elevated AST, normal ALB and HCT); 9.6 (elevated AST, reduced ALB and HCT). * 2. Final model parameter estimates for two periods: Days 0–3: CL/F: 11.1 +/− 12.07 (normal AST, ALB and HCT); 8.04 ± 21.02 (AST >/= 500 U/L or slow recovery); V/F: 328 +/− 21.65 L. Days 4–15: Cl/F: 17.8 ± 6.46 (normal AST, ALB and HCT); 24.5 ± 7.55 (HCT < 28% and ALB < 2.5 g/dL); V/F: 568 ± 2.36 L.

## Data Availability

All data are published in the article.
